# Population level physical activity before and during the first national COVID-19 lockdown: A nationally representative repeat cross-sectional study of 5 years of Active Lives data in England

**DOI:** 10.1016/j.lanepe.2021.100265

**Published:** 2021-11-30

**Authors:** Tessa Strain, Stephen J. Sharp, Andrew Spiers, Helen Price, Ciara Williams, Carol Fraser, Søren Brage, Katrien Wijndaele, Paul Kelly

**Affiliations:** a MRC Epidemiology Unit, University of Cambridge, UK; b Physical Activity for Health Research Centre, University of Edinburgh, UK; c Sport England, London, UK

**Keywords:** Adults, Physical Activity, Covid-19, Surveillance, Domains, CI, Confidence Interval, MR, Mean ratio, OR, Odds ratio

## Abstract

**Background:**

To limit the spread of COVID-19 in March 2020, the population of England was instructed to stay home, leaving only for essential shopping, health-care, work, or exercise. The impact on population activity behaviours is not clear. We describe changes in duration and types of activity undertaken by adults ≥16 years in England between March and May 2016-19 and 2020, by socio-demographic strata.

**Methods:**

Using nationally representative data collected between November 2015 and May 2020 by the Sport England Active Lives Surveys (n=726,257) we assessed trends in amount and type of non-occupational moderate-to-vigorous physical activity. Using data from n=74,430 mid-April to mid-May respondents, we then estimated the odds ratios of reporting any activity in the four-week recall period in 2020 compared to 2016-19. Gamma regressions estimated the mean ratios (MR) of duration amongst those reporting any activity in 2020 compared to 2016-19.

**Findings:**

Population activity declined substantially after the restrictions were introduced. Compared to 2016-19 levels, the odds of reporting any activity in 2020 were 30% lower (95% confidence interval (CI) 26-34%). The largest declines were amongst non-white ethnicities, the youngest and oldest age groups, and the unemployed; no socio-demographic subgroup had higher odds. Amongst those undertaking activity, weekly duration was similar in the two periods (MR 0.99, 95%CI (0.96-1.01%)). The odds of participating in walking for leisure and gardening were 11% (6-16%) and 15% (9-21%) higher, respectively, whereas the odds for team and racket sport and walking for travel participation were 76% (73-79%) and 66% (64-68%) lower, respectively.

**Interpretation:**

Restrictions introduced in Spring 2020 likely reduced physical activity levels in England. The magnitude of the declines were not uniform by demographic groups or by activity type, which future policies should consider.

**Funding:**

TS, KW, SJS, and SB are supported by UK Medical Research Council [grant numbers MC_UU_00006/4 and MC_UU_12015/3] and SB is supported by the NIHR Biomedical Research Centre in Cambridge (IS-BRC-1215-20014).

**Research in Context**

**Evidence before this study**

We searched MEDLINE, Embase, and World of Science for peer-reviewed articles published until June 23 2021 using the following search terms: (COVID-19 or "Novel Coronavirus" or "2019 novel coronavirus" or 2019-nCoV or SARS-CoV-2) AND (“physical activity” or exercise) AND (population or “national surveillance”) AND (isolation or self-isolation or lockdown or lock-down or restriction). We also performed directed searching of databases.

We found 7 peer-reviewed articles investigating changes in physical activity levels concurrent with pandemic-related restrictions in samples with efforts to be nationally representative from Belgium,^14^ France,^15^ Germany,^16^ Saudi Arabia,^17^ the United Kingdom^18^ (birth cohorts), and the United States^19^^,^^20^ (n=362-30,134; see Supplementary Material 1 for further details).

All studies identified individuals and groups with divergent trajectories, some increasing, some maintaining, and some decreasing their activity levels, with a trend towards greater proportions decreasing.

**Added value of this study**

This is the largest nationally representative study of changes in physical activity levels concurrent with pandemic-related restrictions. It is the only one able to control for seasonality and other drivers of natural variation in activity levels by comparing against data from the same time period in the four years previous rather than retrospective recall to the month(s) before restriction were imposed.

Our results indicate that population levels of activity in England declined during the first month of restrictions in 2020, from a relatively stable level in the previous 4 years. No demographic subgroup we investigated had higher levels in 2020 compared to 2016-19 with the largest declines evident amongst non-white ethnicities, younger age groups, and the unemployed.

As the only nationally representative study to date to have asked detailed questions on the types of activities undertaken, we were able to show that the levels of team and racket sports and walking for travel were considerably lower in 2020 compared with 2016-19, whilst gardening and walking for leisure were higher. The relative prevalence of these activity categories amongst demographic subgroups in 2016-19 (e.g. team sports prevalence was lower amongst younger ages and men; gardening prevalence was higher amongst older ages) explain the magnitudes of the declines in overall activity levels.

**Implications of all the available evidence**

Restrictions introduced in Spring 2020 to limit the spread of COVID-19 likely reduced physical activity levels in England. The impacts varied by demographic groups or by activity type, exposing potential underlying inequalities for undertaking and maintaining activity, suggesting that different physical activity support and promotion approaches are needed. Future policies should consider these findings to enhance population opportunities for an active lifestyle.

Alt-text: Unlabelled box

## Introduction

On 23rd March 2020, the UK government introduced a national lockdown to limit the spread of COVID-19 in England.^1^ Individuals were only permitted to leave their homes to shop for necessities, for medical needs, to travel to or from work where absolutely necessary, or for one bout of daily exercise.^1^ All sports facilities and non-essential shops were closed.

There is a mixed picture regarding the effect of lockdown on population physical activity. Immediate data from FitBit users showed lower daily step counts than the equivalent week in 2019 across countries that had imposed stay-at-home orders.^2^ However, Ding et al. (2020) reported increases in the Google Relative Search Rate for exercise-related terms in Australia, the USA, and the UK in the initial period after lockdown imposition.^3^ Data from a rapidly instigated Sport England Savanta ComRes survey (n=2,034) showed 31% reported doing more activity in the first week of April compared to retrospective reporting of activity during the pre-restriction period, whilst 41% reported doing less.^4^ A systematic review investigating changes in physical activity before and during lockdowns found the majority of studies reported decreases, although the quality of included studies was not high.^5^

Physical activity is an important lifestyle behaviour for the prevention of premature mortality and chronic disease, and for promoting mental wellbeing.^6^ As the burden of COVID-19 deaths is falling disproportionately on those with chronic diseases and the obese,^7^ and mental health has deteriorated during the pandemic,^8^ physical activity is of particular relevance at this time. Physical activity may also have positive impacts on the body's immune system,^9^ with some suggesting an increased resilience to severe disease following COVID-19 infection.^10^ Its importance to individual well-being, personal freedom, and population health was underscored by the inclusion of exercise as one of only four reasons for leaving home during the first English lockdown. Understanding how population levels of physical activity changed concurrent with the imposition of the national lockdown, what types of activity people undertook, and any differential changes between demographic groups, will inform policy responses as we emerge from the pandemic.

Sport England's Active Lives Survey offers a unique perspective on this issue as it was the only national physical activity survey in the UK that was able to continue uninterrupted after lockdown due to its remote rather than in-person method of data collection.^11^ With a typical monthly sample size in excess of n=10,000, weighted to be nationally representative, detailed, valid, and reliable population and demographic subgroup comparisons between activity levels in 2020 and previous years can be made. Importantly, these comparisons account for seasonal variations in activity levels and do not rely on retrospective recall to the month(s) before restriction were imposed. In addition, the survey includes a sequential element of broader activity participation for the past 3 months, which allows examination of within-person trajectories.

Initial findings from March-May 2020 have already been published by Sport England, showing a 7.1 percentage-point drop in the proportion of adults meeting the aerobic physical activity guidelines (150 mins of moderate intensity activity or 75 minutes of vigorous or an equivalent combination^12^) in 2020 compared with the same period in 2019. The largest drops occurred amongst men, 16-34 year olds, those with multiple disabilities, and amongst ethnic minority groups.^13^ However, using a dichotomous metric of physical activity guideline compliance may miss important changes across the wider distribution and composition of activity levels. This study aims to provide greater insight into the way physical activity levels changed during the first COVID-19 lockdown in England.

The first objective of this study was to provide the five-year context for activity levels of March-April 2020 by presenting seasonal trends and the breakdown of activity by type. The second objective was to estimate the differences in physical activity levels between March-April 2020 and the corresponding seasonal period in the pre-Covid years (2016-19), stratified by socio-demographic subgroups. The third objective was to investigate within-person changes in physical activity in a subset of the sample over the 3-month period preceding the Spring 2020 lockdown, again comparing to pre-Covid years.

## Methods

### Data source and sample

The Active Lives Survey is a nationally representative survey of the leisure-time and travel-related physical activity behaviours of adults in England aged ≥16 years.^11^ It has run continuously since mid-November 2015 in its current form, achieving an annual sample size >170,000 through random probability sampling. It is a ‘push-to-web’ survey design, meaning potential participants are sent a letter containing information about how to access the survey online, either on a mobile, tablet, or PC/laptop. A paper version is sent alongside a third (maximum of four) prompt letter.

We excluded individuals <16 years or if they completed the young person's questionnaire (2015/6 and 2016/7 only) or were missing age data, were missing the appropriate weighting variable or covariate data, or reported implausible levels of activity (Supplementary Figure 1). The remaining participants were included in the overview of activity levels from 2015-2020. For the analysis of the impact of lockdown, we only included mid-April to mid-May respondents. That sample was further reduced to online respondents for the within-person analyses as the relevant questions were not in the paper version.

### PA data collection

In both the online and paper versions of the questionnaire, respondents reported the frequency, average duration, and intensity of physical activities they participated in over the previous 4 weeks.^11^ A range of team and individual sports, exercise and fitness activities, walking and cycling for travel and leisure, and gardening were prompted, with the opportunity to add similar non-listed activities. Only walking specified a minimum bout length of 10 mins, an instruction that was removed in November 2019. In the online version, prior to reporting on the past 4 weeks, respondents indicate whether they participated in the activity in the previous 3 months.

### Physical activity summary variables

Average weekly duration (minutes/week) in each activity was calculated by multiplying the reported frequency and duration during the four-week recall period and dividing by four. In the main analyses, only activities that were considered by Sport England analysis protocols to be moderate-to-vigorous were included. Intensity level classification was determined primarily by reference to the Compendium of Activities MET values with the responses to the questions on markers of relative intensity resolving activities that could span intensity categories on an individual basis.^21^ Only activities with a reported duration of >10 minutes were included. Duration of vigorous intensity activities was doubled to reflect the additional health benefits compared to moderate activity, as per the UK physical activity guidelines.^12^ The main outcome variable was therefore weekly moderate intensity-equivalent duration of activity. We excluded those reporting implausible values (>6720 moderate-equivalent total non-occupational minutes/week (i.e. >16 hours/day); <0.5% sample, Supplementary Figure 1).

Eleven mutually exclusive subgroups of activities (walking for leisure, walking for travel, cycling for leisure and sport, cycling for travel, team and racket sports, golf, fitness activities, running/jogging/athletics and multi-sports, swimming and diving, other sports and leisure activities, gardening) were derived (see Supplementary Material 2).

### Within-person changes in physical activity

Within-person changes were derived from the responses to the questions on participation in the 3 months and 4 weeks prior to interview. Due to the overlapping time-periods, if a respondent confirmed they did not participate in the last 3 months, they were not asked any questions about the last 4 weeks (see Supplementary Figure 2). If a respondent did participate in the last 3 months, they were then asked to confirm whether they had participated in the last 4 weeks. Respondents were categorised as: (1) no participation in the 3 months prior to interview, (2) participation in the previous 4 weeks (it was not possible to determine if these individuals also participated in the 2-3 months prior to interview), and (3) participated in the 2-3 months prior to interview but not in previous 4 weeks. Activity participation included light intensity activity and had no minimum bout duration.

### Demographic and geographic variables

We identified demographic and geographic variables that were plausibly related to changes in physical activity levels during the first Covid-19 lockdown. These included: gender (men, women, other), age (16-24, 25-34, 35-44, 45-54, 55-64, 65-74, 75-84, 85+ years), ethnicity (White British, White Other, Black, Asian (excluding Chinese), Chinese, Mixed, Other Ethnic Group), disability status (no disability, non-limiting disability, limiting disability), work status (working full or part time, unemployed, retired, student full or part time, other working status), Index of Multiple Deprivation (deciles), NS SEC measure of social class (higher social groups, middle social groups, lower social groups, students and other unclassified, out of age range >75 years), age of child(ren) in the household (three separate binary variables for child(ren) aged under 5 years, 5-10 years, and 11-15 years), region (London, East Midlands, East, North East, North West, South East, South West, West Midlands, Yorkshire and the Humber), urban-rural (major urban conurbation, minor urban conurbation, urban city and town, rural town, rural village, rural hamlet), household living arrangements (couple with child(ren) ≤15 years in household, couple with no children ≤15 years in household, lone parent with child(ren) ≤15 years in household, single person living alone, other/complex including multi-generational households), and body mass index category (BMI; normal and underweight <25 kg/m^2^, overweight 25-30kg/m^2^, >30 kg/m^2^). Data on household living arrangements were collected from November 2016 onwards.

### Statistical analyses

Weighted mean and median duration of moderate-equivalent activity by month of response were examined to ascertain the inherent seasonal patterns in activity behaviours amongst respondents from November 2015 - May 2020 (n=726,257). We also investigated how the contribution of the eleven mutually exclusive activity types varied across this period.

Next, using the mid-April to mid-May samples (n=74,430), we compared the activity levels of this same time period in 2020 with 2016-19. The 4-week recall period for the majority of 2020 respondents fell entirely in lockdown and this analytical approach allowed us effective control for seasonality. As total non-occupational physical activity approximated a zero-inflated gamma distribution, we used two models (logistic and gamma regression) to estimate the differences in physical activity levels between the 2016-19 and 2020, stratified by socio-demographic subgroups. Logistic regression estimated the odds ratios (ORs) of reporting >0 mins/week and gamma regression estimated the mean ratio (MR) of weekly duration amongst those reporting >0 mins/week for 2020 relative to 2016-19. We calculated age- and sex-adjusted estimates and multivariable-adjusted estimates, the latter including all other socio-demographic variables as potential confounders. The exceptions to this were household living arrangements, as this was not asked in the 2015/16 survey, and BMI, due to extensive (weighted n=11,671) missing self-reported height and weight data. We undertook sensitivity analyses including household living arrangements as an additional covariate, restricting the data to 2016/17 onwards. We also undertook investigations into the addition of BMI as a covariate, and the change in activity levels within those with missing BMI and other covariate data.

We focus on the results for the whole sample and sex, age, ethnicity, working status and disability status subgroups. This decision was made based on important inequality characteristics and inspection of the results; all other subgroup analyses are available in supplements. We repeated the analyses for specific activity types that had a combination of sufficiently high prevalence and large differences between 2016-19 and 2020 to provide meaningful analyses.

For the analysis of within-person changes in activity before and during lockdown (n=49,073 online respondents), we compared the proportions reporting no participation in the 3 months prior to interview, participation in the previous 4 weeks, and participated in the 2-3 months prior to interview but not in previous 4 weeks, in 2020 with those in 2016-19.

All analyses were weighted to account for selection probabilities and non-response at a monthly sample level, calibrated to age, gender, household size, long-term health problems, NS-SEC, and highest educational qualification population totals within local authority areas.^21^ Variance was adjusted for local-authority level stratification. Analyses were undertaken in Stata v.16 (StataCorp, TX, USA) and figures were produced in R and Biorender.

### Role of funding source

The funders had no role in study design, data collection, data analysis, interpretation or writing of the report.

## Results

### Overview from 2015-2020

**Figure 1:**
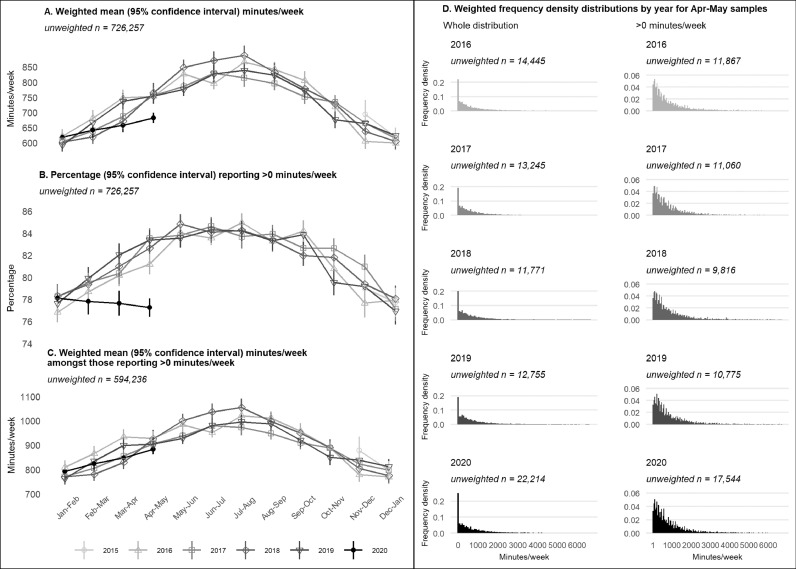
Non-occupational physical activity weekly duration (moderate intensity-equivalent) by month of response from November 2015 to May 2020, (n=726,257).

There were n=726,257 respondents (monthly range n=4,909-23,839) from November 2015 to May 2020. The mean weekly duration of moderate intensity-equivalent activity for April-May 2020 respondents was the lowest for that month across all survey years (682 mins/week in 2020 versus 754-765 mins/week in 2016-2019; Figure 1, Supplementary Table 1). The proportion of people reporting any activity (>0 mins/week) was lowest (77.2% in 2020 versus 81.2-83.5% in 2016-2019), and the mean weekly duration amongst those reporting any activity was at the lower end of the observed range (882 mins/week in 2020 versus 904-929 mins/week in 2016-2019).

**Figure 2:**
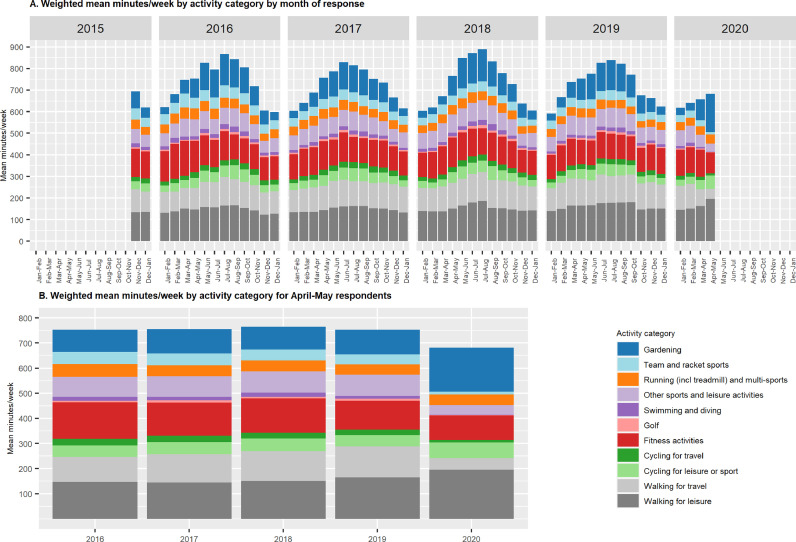
Composition of mean non-occupational physical activity (moderate intensity-equivalent) weekly duration by activity category from November 2015 to May 2020, (n=726,257).

Figure 2 shows how the composition of different activities varied across the period. The lower mean overall physical activity levels in 2020 (682 mins/week) can be clearly seen. Mean weekly duration of gardening and walking for leisure were higher in 2020 than any of the previous four years (176 and 196 mins/week in 2020 versus 89-99 and 144-165 mins/week in 2016-19, respectively; Supplementary Table 2). Mean weekly duration of all other activity categories were lower in 2020 than the previous four years resulting in overall lower activity levels.

### Total non-occupational activity in April-May

The socio-demographic characteristics for the n=74,430 April-May respondents are presented in Supplementary Table 3 and a comparison between the sample with and without those missing covariate data is shown in Supplementary Table 4.

**Figure 3:**
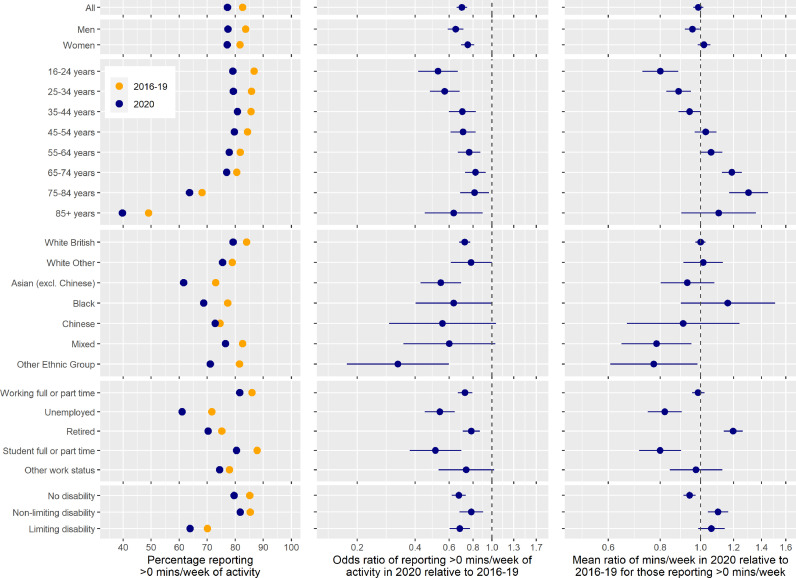
Comparisons of total non-occupational physical activity levels in April-May respondents in 2016-19 and 2020, by selected demographic subgroups, (n=74,430).

Figure 3 shows the percentage reporting any activity (>0 mins/week) of total non-occupational physical activity in 2016-19 and 2020, the age/sex and multivariable-adjusted ORs for reporting any activity in 2020 relative to 2016-19, and the age/sex and multivariable-adjusted MRs of weekly duration for those reporting >0 mins/week in 2020 relative to 2016-19 for selected socio-demographic subgroups. Data for all socio-demographic subgroups are in Supplementary Table 5.

*Odds and mean ratios adjusted for age, sex, ethnicity, working status, disability status, NS SEC social class, education level, deprivation decile, children under 5 years in household, children 5-10 years in household, children 10-15 years in household, region, and urban-rural location. Analysis sample for mean ratios only include those reporting >0 mins/week.*

The odds of reporting any activity from the multivariable-adjusted model were 30% lower (26-34%) in 2020 compared to 2016-19 for the whole population. The largest reductions in odds of reporting any activity were seen amongst non-white ethnicities, younger age groups, the unemployed, and students. For example, those of Asian (excluding Chinese) ethnicity were 46% (31-57%). There was greater variation in the activity duration for those reporting >0 mins/week in 2020 relative to 2016-19 by subgroup. The MRs for weekly duration were below 1 for younger adults, the unemployed, and students denoting shorter durations of activity in 2020 relative to 2016-19 amongst those doing the activity. In contrast, MRs were above 1 for retired/55-84 year olds (e.g. 1.30 (1.17-1.45) in 75-84 year olds).

Additional adjustment for household living arrangements did not materially change the results with the exception of Black, Chinese and Other Ethnic Group ethnicities where the OR of reporting any activity were all strengthened by over 10 percentage points (Supplementary Table 6). Additional adjustment for BMI generally attenuated the ORs slightly (Supplementary Table 6), although this analysis excludes those with missing BMI data (weighted n=11,671) who showed a greater differential in activity levels between 2016-19 and 2020 than those in other BMI strata (Supplementary Table 7).

### Activity types

The composition of mean total non-occupational activity by activity type amongst the April-May respondents for 2016 to 2020 is shown by gender, age, ethnicity, work and disability status in Supplementary Figures 3A-E. Men, younger adults, and students all had relatively higher levels of team sport compared to other demographic groups in 2016-19 but this almost disappeared in April-May 2020. Gardening was already a more prevalent activity in older age groups in 2016-19, and in 2020 levels increased, mitigating the overall impact of lower levels in other activity types for this age group. Levels of walking for leisure were almost universally highest in 2020 amongst all subgroups, although a slight increasing trend was already evident since 2016. Meanwhile, walking for travel was lower across almost all groups in 2020, with younger age groups/students (who had the highest levels in 2016-19) showing the greatest differences.

Supplementary Figures 4A-E and Supplementary Tables 8-12 show the comparisons for walking for leisure, walking for travel, gardening, fitness activities, and team and racket sports. All subgroups except those over 75 years, Chinese ethnicity, Other Ethnic Group, and the unemployed were more likely to report any walking for leisure and those reporting >0 mins/week were more likely to report higher durations in 2020 compared to 2016-19. In contrast, all subgroups were less likely to report walking for travel with most multivariate ORs comparing 2020 to 2016-19 falling in the 0.2-0.4 range. However, the MR amongst those reporting >0 mins/week did differ for most subgroups, the exceptions being the youngest and oldest age groups, students, the unemployed and those in lower social groups where decreases in reporting durations were evident. The ORs for reporting >0 mins/week of gardening in 2020 compared to 2016-19 were higher in the younger and older compared to the middle age groups, despite very differing prevalence levels. MRs for the durations amongst those reporting >0 mins/week were almost universally higher across subgroups, mainly in the range of 1.4-2.0.

### Within-person changes in participation

**Figure 4:**
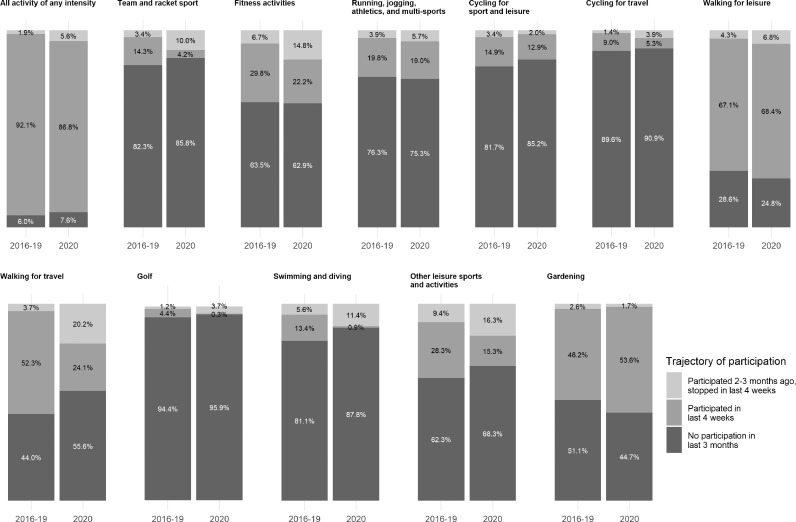
Within-person trajectories of participation for the April-May samples in 2016-19 and 2020, (n=49,073).

There were two indicators that activity participation was lower in mid-March to mid-April in 2020 than in 2016-19 (Figure 4; Supplementary Table 13). Firstly, there was a greater proportion of respondents who stopped participating in any activity of any type in the month prior to interview in 2020 (5.6% (5.0-6.1%)) versus 2016-19 (1.9% (1.7-2.1%)). Secondly, in the three months prior period, which includes the lockdown month, there was a higher proportion not doing any activity in 2020 versus 2016-19 (7.6% (7.0-8.3%) and 6.0% (5.6-6.4%), respectively).

*Footnote. It is not possible to directly identify individuals who do not do an activity 2-3 months ago and then take up that activity in the last 4 weeks.*

The greatest differentials in the proportions stopping activity in the last month were seen in fitness activities (14.8% (14.1-15.6%) versus 6.7% (6.3-7.1%)) and walking for travel (20.2% (19.3-21.2%) versus 3.7% (3.5-4.0%)). There was a higher proportion stopping walking for leisure in the last month in 2020 compared with 2016-19 (6.8% (6.2-7.3%) versus 4.3% (4.0-4.6%)), but the proportion not participating at all in the last three months was lower (24.8% (23.8-25.8%) versus 28.6% (27.9-29.4%)).

As sensitivity analyses to investigate fluctuations in participation amongst those who participated in activity in the last month, we looked at additional data on participation levels in the 4-12 months prior to interview. We found that approximately 85 of the 90% that participated in any activity the last three months had also participated at all earlier time points in both 2016-19 and 2020.

## Discussion

In this large sample representative of the England population, we show that lockdown restrictions in April-May 2020 likely reduced physical activity participation from 83% to 77% (representing 30% lower multivariable-adjusted odds) compared to the same time period in previous years. Mean weekly physical activity duration was around one hour lower in 2020 than previous years, a 10% reduction. Some subgroups showed larger declines than others, most notably non-white ethnicities, the youngest and oldest age groups, and the unemployed. In those that reported any activity, there was no difference in mean weekly duration of activity at a population level

The overall level and composition of physical activity types displays a distinct seasonal pattern, being higher in the summer months than winter months. Taking advantage of the large sample and using seasonal matching, we were able to account for these underlying temporal patterns when examining the potential impact of lockdown restrictions on population activity levels.

The time trends in activity type composition confirms near-complete disappearance of team sports, facility-based activity and walking or cycling for travel in 2020. This is consistent with purported impacts of lockdown rules designed to facilitate social distancing. In contrast, levels of gardening, walking for leisure and cycling for leisure were higher compared to the previous four years, however total walking levels (leisure and travel) were still substantially reduced in 2020. Although the within-person analyses indicated a higher proportion stopped walking for leisure in first month of lockdown, this is not necessarily contradictory. It may reflect a wider increasing trend in participation meaning there was a greater proportion able to stop participating in 2020.

The differences between 2016-19 and 2020 were slightly larger in men than women, and in younger age groups than middle-aged, which may relate to the way lockdown impacted different activity types. The oldest age groups showed a decline in participation of similar magnitude comparable to the youngest age groups, however, the MRs were more ambiguous regarding changes in mean durations. A consequence of this undesirable change in activity level was an attenuation in these particular demographic inequalities, although men and younger age groups were overall still more active in 2020. The maintenance, and in some cases, increase in cycling for leisure and sport and gardening appear key in compensating for the loss in organised sport for these groups. Conversely, some existing inequalities were exacerbated with differences increasing between 2016-19 and 2020 amongst those of Asian (excluding Chinese) and Black ethnicities, those over 85 years, and those who were unemployed. Whilst there were large relative increases in the proportions participating in gardening amongst some of these groups, the absolute levels remained lower and were insufficient to compensate for declines elsewhere. Additionally, walking for leisure was another activity type that was higher at a population level in 2020, but not consistently for the subgroups identified. Differences were relatively uniform across disability status. By comparison, data from the USA suggested an overall increase in activity participation since the pandemic but a widening in inequalities between the sexes and different ethnic groups.^19^

There have been various analyses of walking and cycling levels during the COVID-19 pandemic, with suggestions these activity types increased,^22^^,^^23^ including an analysis of smartphone step count data in US cities that suggested a notable decrease in utilitarian walking, whilst leisure walking was less affected.^24^ These results by type of activity may help explain differences seen by demographic group; odds ratios of reporting any activity were lower in non-white ethnicities, younger age groups, the unemployed, students and those experiencing more deprivation. These groups may have been more reliant on the types of activities most affected by lockdown. In addition, they may have had less access or opportunity to walk for leisure or garden, two primary types of activity that increased.

These nationally representative results support initial indications that the 2020 lockdown was associated with a net reduction in physical activity levels at the population level in England.^3^^,^^4^ Some early studies from the UK suggested notable proportions of individuals increasing their physical activity.^25^^,^^26^ These studies had non-representative samples, skewed to affluent, middle-aged or retired, or female groups. Indeed, our analyses showed that amongst those that reported any activity, the durations of that activity increased amongst the middle-to-older age groups. However, it is difficult to ascertain from these data whether these increases were due to individuals increasing their activity durations or if those previously undertaking only short durations of activity stopped participating thus shifting the average higher. In any case, it is likely there was significant heterogeneity of the change in physical activity levels in response to lockdown which our group-level analyses are unable to detect. Changes in employment status (e.g. furlough), location and demands, individual attitudes and motivations, amongst other factors, are plausible candidates for interaction with the demographic and geographical characteristics we investigated.

There are a number of important strengths and limitations of the results reported here. In contrast to previous reporting on the impacts of lockdown restrictions in England, these data are based on a large sample size of over 70,000 seasonally matched respondents, designed to be nationally representative which minimises selection/sample bias, using a repeat cross-sectional design rather than long-term retrospective recall to infer change. This accounts for seasonal variation, and as our data show, should be borne in mind when interpreting the results of other studies comparing activity levels from different periods. The sample size also allows investigation by important demographic groups and certain protected characteristics making the findings more policy relevant.^27^ Further, the detailed information on activity type allowed for the identification of differences in key behaviours such as walking for leisure, active travel, gardening, and organised sport, which provides essential and complimentary information for policy and health promotion, compared to just overall levels. Finally, the analysis of within-person data allowed for more direct inference on change in behavior from the months leading up to lockdown in 2020, when contrasted against such within-person trajectories in previous years (2016-19). These results corroborate the evidence from the repeat cross-sectional data, supporting the overall assertion that lockdown negatively impacted on population levels of activity.

There are a number of limitations to consider as well. A key assumption is that the samples are population representative at each time-point, and that differences in activity behaviours are not a result of differential sample bias. It is possible that factors such as key worker or furlough status may have affected the probability of survey participation in 2020 but were not fully accounted for in the sample weighting. However, given the comparable proportions of respondents across a large range of socio-demographic subgroups across the survey years, this is not likely to have materially influenced the results. Measurement of such factors would also have been useful from a subgroup analysis point of view; it is possible that despite the range of socio-demographic variables, other factors were critical to determine the change in activity levels in 2020. Another issue is the self-reported nature of the data, which may introduce recall bias. If this bias varied by survey year, demographic subgroup or activity type, this could be influential on the conclusions. However, this assessment method is also what allowed such a large sample size and assessment of such a range of behaviour types, and should be considered the appropriate method until it is feasible to complement it with device-based assessment of activity at this scale.^28^ The main analyses focused on moderate-to-vigorous physical activity as this is relevant to the main indicator for adult physical activity prevalence in England, even though light intensity is now also part of the UK CMO Guidelines for Physical Activity.^12^ It is possible that light intensity behaviours are where the greatest changes have occurred, at least for certain demographic groups. However, durations of light intensity activity are hard to recall accurately given their frequent but intermittent nature, and are often undertaken as part of work or housework, domains that were out of scope for the Active Lives survey. Finally, the configuration of the within-person participation trajectory questions prevented the identification of those that initiated an activity in the last month. This was lower for seasonal activities like gardening, but was not suggestive of a large proportion of the population taking up a new activity in March-April 2020.

There are a number of implications from these analyses. Lower activity levels are likely to result in detrimental physical and mental health. As an example, a recent analysis suggests that the number of falls could increase by around 5% in England due to deconditioning amongst older adults.^29^ Given the pressure on the health system in England with the large backlog in routine healthcare, healthy lifestyles for primary and secondary prevention become even more important. In addition, the larger differences evident amongst lower social classes and the unemployed may exacerbate already existing inequalities,^27^ heightening the policy priorities of supporting physical activity and well-being in these groups.^30^ However, it has been suggested that the mechanisms between disease and social factors, particularly in the context of COVID-19, are not well understood,^30^ and that new ways of conceptualising the challenge will be required to address it.

Another possible implication is that we should support participation across multiple and varied physical activity types to increase behavioural resilience to pandemic restrictions or other life disruptions. If people rely on a single or limited source of physical activity, they may be more vulnerable to changes in circumstance.

In this specific study, the increases in leisure walking and cycling among certain groups may give cause for optimism in future physical activity promotion, indicating that positive population shifts are possible if people have time and access to attractive spaces in which to be active. The implications for policy and delivery are perhaps self-evident.

Overall, the varied impacts by demographic group may support the notion that certain groups of people respond differently to intervention for different physical activity behaviours. This suggests that a broad range of physical activity support and promotion approaches are needed at a population level,^31^^,^^32^ as opposed to a one-size-fits-all approach. This may apply both to maintaining and increasing population levels of activity, as well as limiting their decline in periods of crisis.

In terms of future research recommendations, the present analyses only assess the first month of lockdown. It will also be interesting to monitor how activity patterns change as restrictions evolve and get lifted, including during future pandemics. For example, will sport activity rebound in young people? Will active travel patterns be impacted long-term by changes in working from home? This latter question is another that may show demographic differences based on nature of work. These analyses use traditional approaches to assessing inequality. More sensitive and fine-tuned measures that reflect 21^st^ century society are needed to better understand demographic impacts, and by extension identify relevant solutions. The pandemic may have created new (or exacerbated existing) inequalities such as access to green space or garden, or key worker status, details of which should be investigated. Such data are not currently collected in the Active Lives Survey, but questions on green space were added to Sport England's Children and Young People's Survey^33^ and their Savanta ComRes tracker survey^4^ to allow further research on this issue. Indeed, the scoping review by Yomoda et al. (2021) highlights the importance of researching the effect of the pandemic on children's physical activity levels.^34^

The COVID-19 pandemic-related lockdown may have reduced population levels of physical activity in England in the Spring of 2020. However, the impacts were not uniform and varied by demographic groups and by activity type, exposing potential underlying inequalities for the ability to undertake and maintain activity. Future policies should be mindful of these findings to enhance population opportunities for an active lifestyle, by considering the importance of activity type, and the differential effects across demographic groups.

## Data Sharing

Data are available on the UK Data Archive.^11^

## Contribution statement

Conceptualisation/study design (all authors), data curation (HP, CW), formal analysis (TS), methodology (SB, SJS, KW, PK, TS), writing - original draft (TS, PK), writing - reviewing & editing (all authors). HP and CW verified the initial dataset. TS takes responsibility for the analysis.

## Declaration of Interests

AS, HP, CW, and CF are employees of Sport England, an arms-length body of government responsible for growing and developing grassroots sport and getting more people active across England. It is funded by the UK Government and the National Lottery.

TS, KW, SJS, and SB are supported by UK Medical Research Council [grant numbers MC_UU_00006/4 and MC_UU_12015/3] and SB is supported by the NIHR Biomedical Research Centre in Cambridge (IS-BRC-1215-20014).

PK and TS were awarded a contract for statistical services related to quality assurance on Active Lives Survey (Aug 2019 – Aug 2021).

SB and TS were awarded honorariums for speaking at a symposium organised by Yonsei University, Korea in Aug 2021. TS was remunerated by the Arctic University of Norway for assessing a PhD defence. TS co-chaired the UK Chief Medical Officers` Expert Group on Physical Activity Surveillance (Sept 2019 – Aug 2021).

## Appendix Supplementary materials

Image, application 1

Image, application 2

## Footnotes

Supplementary material associated with this article can be found, in the online version, at doi:10.1016/j.lanepe.2021.100265.
