# Supplementary material for: Population level physical activity before and during the first national COVID-19 lockdown: A nationally representative repeat cross-sectional study of 5 years of Active Lives data in England
Source: Lancet Reg Health Eur. 2021 Nov 30;12:100265. doi: 10.1016/j.lanepe.2021.100265 (PMC8629728; doi:10.1016/j.lanepe.2021.100265)
Supplement: Supplementary file 2 [file mmc2.docx]

**Supplementary Material 1. Summary of studies on changes in physical activity level associated with Covid-19 lockdowns using sampling methods and weighting to obtain nationally or regionally representative samples.**

Bann D, Villadsen A, Maddock J, Hughes A, Ploubidis GB, Silverwood R, et al. Changes in the behavioural determinants of health during the COVID-19 pandemic: gender, socioeconomic and ethnic inequalities in five British cohort studies. J Epidemiol Community Health. 2021

Sample: n=13,997 from five nationally representative British/English birth cohorts (birth years 1946, 1958, 1970, 1989-1990 and 2000-2002).

Fieldwork: May 2020, online questionnaire.

Physical activity measure: Number of days per week that participants exercise for 30 min or more at moderate-to-vigorous intensity (“working hard enough to raise your heart rate and break into a sweat”) in the month before fieldwork. Retrospective recall to “the month before the Coronarvirus outbreak”.

Findings: Mean number of days per week was higher during the pandemic that pre-pandemic for all birth cohorts (range across cohorts pre-pandemic 2.7-3.3 days/week, during pandemic: 2.9-3.5 days/week). Changes occurred in both directions with shifts away from the middle of the distribution to the extremes evident. Previous inequalities between men and women were nullified during the pandemic as relatively more women reported increased levels. Socio-economic inequalities were evident before and during the pandemic whilst ethnic inequalities became apparent during the pandemic.

BinDhim NF, Althumiri NA, Basyouni MH, AlMousa N, AlJuwaysim MF, Alhakbani A, et al. Exploring the Impact of COVID-19 Response on Population Health in Saudi Arabia: Results from the “Sharik” Health Indicators Surveillance System during 2020. Int J Environ Res Public Health. 2021;18(10).

Sample: n=30,134 of Arabic speaking Saudi residents aged ≥18 years were recruited via proportional quota sampling to obtain an equal distribution of participants (stratified by age and gender) within and across the 13 administrative regions of Saudi Arabia.

Fieldwork: Repeat cross-sectional, 7 waves of data collection from January-December 2020 summarised into quarters, telephone questionnaire.

Physical activity measure: Based on responses to duration, frequency and intensity of physical activity, respondents were categorised as meeting the WHO guidelines or not.

Findings: Compared to Q1, multivariable-adjusted odds ratios of meeting the guidelines were lower in Q2-Q4 (0.48-0.53).

Constandt B, Thibaut E, De Bosscher V, Scheerder J, Ricour M, Willem A. Exercising in Times of Lockdown: An Analysis of the Impact of COVID-19 on Levels and Patterns of Exercise among Adults in Belgium. Int J Environ Res Public Health. 2020 Jun 10;17(11).

Sample: n=13,515 of Flemish (Belgian) citizens aged ≥18 years recruited via local newspaper, public and private sports organisations, social media, and email. Data were weighted in terms of sex, age, highest educational level, and household composition to be representative of the target population. High active people were strongly overrepresented in the sample and so results were stratified by high and low active.

Fieldwork: March-April 2020, online questionnaire.

Physical activity measure: Respondents were asked whether they were exercising less, as much, or more during the lockdown.

Findings: Amongst the high active, 36% reported doing more than before, 41% as much, and 23% less. Amongst the low active, 58% were exercising more, 5% as much, and 7% less.

Constant A, Conserve DF, Gallopel-Morvan K, Raude J. Socio-Cognitive Factors Associated With Lifestyle Changes in Response to the COVID-19 Epidemic in the General Population: Results From a Cross-Sectional Study in France. Front Psychol. 2020;11:579460.

Sample: n=4005 adults ≥18 years in France recruited from a panel of regular survey respondents, enrolled on a stratified sampling method to reflect the age, sex, occupation and regional distribution of the French general population.

Fieldwork: April 2020, online questionnaire.

Physical activity measure: Respondents were asked whether they were exercising less, as much as, or more during the lockdown.

Findings: 45% of the sample reported exercising less, 43% were unchanged, and 11% reported more.

Mutz M, Gerke M. Sport and exercise in times of self-quarantine: How Germans changed their behaviour at the beginning of the Covid-19 pandemic. Int Rev Sociol Sport. 2021 May;56(3):305–16.

Sample: n=1001 residents of Germany aged ≥14 years were recruited to be representative of the target population.

Fieldwork: March-April 2020, online questionnaire.

Physical activity measure: Respondents were asked how much time they spent playing sport or exercise in their leisure time with the response categories in the previous week and in a normal week before measures of containment were put in place: Did not play exercise or sports, Less than 1 hour, About 1 hour, About 2 hours, About 3-4 hours, About 5-6 hours, About 7-14 hours, 15 hours or more.

Findings: The proportion reporting that they did not play exercise or sports was 39.4% pre-pandemic and 59.5% during the pandemic. The proportions reporting 2 hours, 3-4 hours and 5-6 hours were all lower during the pandemic than pre-pandemic. 48.7% reduced their activity, 42.3% remained the same, 9.0% increased. The proportions reducing their activity were higher in older compared to younger age groups.

Sher C, Wu C. Who Stays Physically Active during COVID-19? Inequality and Exercise Patterns in the United States. Socius Sociol Res Dyn World. 2021 Jan;7:237802312098771.

Sample: n=7,976 adults already part of a probability-based internet panel survey. Sample weights were derived to align the sample with population distributions of gender, race/ethnicity, age, education and geographic location.

Fieldwork: March-December 2020, online questionnaire.

Physical activity measure: Number of days per week on which respondents exercised.

Findings: The mean days per week of exercise increased in the sample from 2.06 in March to 3.7 in April and remained relatively stable for the rest of the data collection period. Existing inequalities between males and females, different ethnicities, income and education levels widened despite all groups reporting a higher number of days per week from April onwards compared to March.

Weaver RH, Jackson A, Lanigan J, Power TG, Anderson A, Cox AE, et al. Health Behaviors at the Onset of the COVID-19 Pandemic. Am J Health Behav. 2021 Jan;45(1):44–61.

Sample: n=362 English-speaking adults ≥18 years living in the United States, contacted online. The sample was cross-stratified on age, ethnicity, and sex to be representative of the population.

Fieldwork: April-May 2020, online questionnaire.

Physical activity measure: The International Physical Activity Questionnaire Short-Form was administered and scored using the protocol to derive MET-minutes/week of activity undertaken in the previous 7 days. Pre-pandemic levels were retrospectively recalled.

Findings: Mean MET-minutes/week decreased from 2205 (SD 3343) to 1616 (SD 2177).

**Supplementary Figure 1. Flowchart of participant exclusions.**


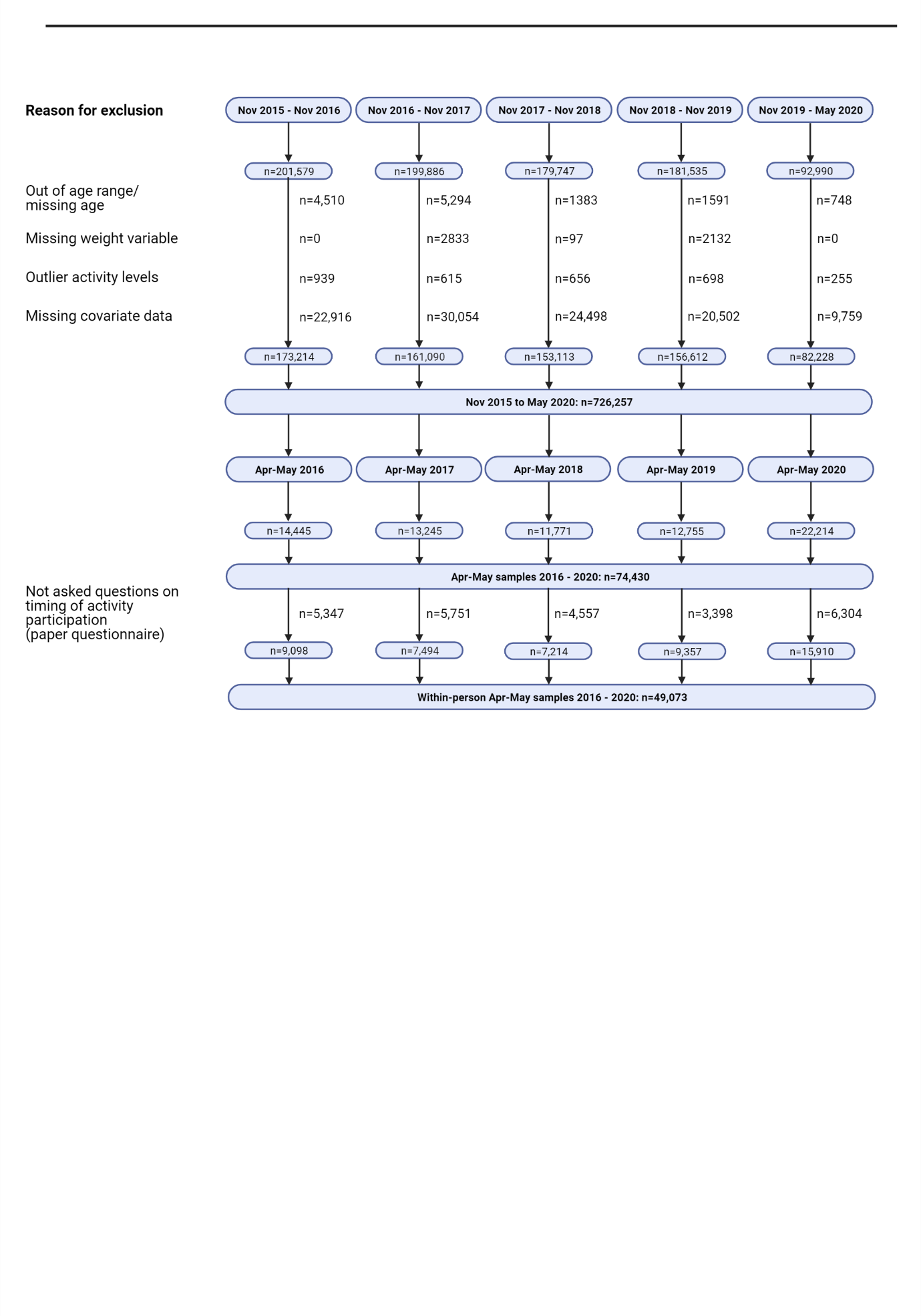


Created with BioRender.com

**Supplementary Material 2. List of sport categories and Active Lives variable names**

The variable names in the Active Lives datasets are in italics. Multiple variable names for similar activities reflect changes in the data coding over the years rather than in the participant-facing questionnaire or duplication.

**Walking for leisure:** Walking for leisure (includes rambling and nordic walking) (*Walk1_2, A1_4_8*), Rambling or nordic walking (*Walk1_3, A1_3_2)*, Walking excluding travel (*Walk1_6*),

**Walking for travel:** Walking for travel (*Walk1_1*)

**Cycle for leisure and sport:** Cycling for leisure *(Cyc1_2),* Mountain biking *(Cyc1_3)*, BMX *(Cyc1_4),* Road cycling or racing *(Cyc1_5)*, Track cycling *(Cyc1_6),* Cyclo-cross *(Cyc1_7)*, Cycling for Leisure and all other cycling *(Cyc1_10)*, Cycling for leisure *(A1_4_9)*

**Cycle for travel:** Cycling for travel incl commuting (*Cyc1*), Cycling for travel (including commuting) (*Cyc1_1*)

**Team & racket sports:** Football *(A1_1_1),* 11 a-side football *(A1_1_1_1)*, Small sided football *(A1_1_1_2)*, Futsal *(A1_1_1_3)*, Walking football *(A1_1_1_4)*, Other football *(A1_1_1_5)*, Hockey *(A1_1_10)*, Field hockey *(A1_1_10_1)*, Roller hockey *(A1_1_10_2, A1_13_2)*, Ice hockey *(A1_1_10_3, A1_6_4)*, Volleyball *(A1_1_11)*, Rounders *(A1_1_12, A1_4_6)*, Dodgeball *(A1_1_13)*, Baseball or softball *(A1_1_14)*, Lacrosse *(A1_1_15)*, Goalball *(A1_1_16)*, Handball *(A1_1_17)*, Gaelic sports *(A1_1_18)*, Cricket *(A1_1_2)*, Long form cricket match (e.g. 40-50 overs) *(A1_1_2_1)*, Short form cricket match (e.g. 20:20) *(A1_1_2_2)*, Other cricket *(A1_1_2_5)*, Cricket nets (*A1_1_2_3, A1_1_2_4,* *A1_1_2_6)*, Rugby union *(A1_1_3)*, 15 a-side rugby union *(A1_1_3_1)*, Touch rugby union *(A1_1_3_2)*, Rugby sevens *(A1_1_3_3)*, Tag or other rugby union *(A1_1_3_4)*, Rugby league *(A1_1_4)*, 13 a-side rugby league *(A1_1_4_1)*, Touch rugby league *(A1_1_4_2)*, Tag or other rugby league *(A1_1_4_3)*, Touch rugby *(A1_1_5)*, Touch rugby union *(A1_1_5_1)*, Touch rugby league *(A1_1_5_2)*, Wheelchair rugby *(A1_1_6)*, Netball *(A1_1_7)*, Basketball *(A1_1_8)*, Wheelchair basketball *(A1_1_9)*, Other team sport *(A1_1_95)*, American football *(A1_1_95_1)*, Korfball *(A1_1_95_2)*, Stoolball *(A1_1_95_3)*, Water polo *(A1_1_95_4, A1_7_6),* Tennis *(A1_2_1)*, Badminton *(A1_2_2)*, Squash *(A1_2_3)*, Racketball *(A1_2_4)*, Table Tennis *(A1_2_5)*, Squash or racketball *(A1_2_6)*

**Golf:** Golf *(A1_9)*, Full course golf *(A1_9_1)*, Short course golf, par 3, pitch and putt, putting *(A1_9_2)*, Driving range *(A1_9_3)*, Adventure or crazy golf *(A1_9_4)*

**Exercise and fitness:** Cycle class (e.g. spinning RPM) *(A2_1_1, A2_5_16),* Core strength class (e.g. legs bums and tums body balance) *(A2_1_12, A2_5_15)*, Boxing class (e.g. Boxercise body combat) *(A2_1_13, A2_5_13)*, Boxing fitness at home *(A2_3_8)*, Weights-based class (e.g. body pump kettlebell) *(A2_1_14, A2_5_20),* Water-based class (e.g. aquaerobics aquafit) *(A2_1_15, A2_5_19)*, Bootcamp (e.g. drill sergeant military fitness) *(A2_1_16, A2_1_18, A2_4_7, A2_4_8, A2_5_9)*, Cross fit *(A2_1_17, A2_4_10, A2_4_5, A2_5_10)*, Dance-based class (e.g. Zumba, fitsteps, ravercise or body jam) *(Dance_2, Dance_3, A2_1_19, A2_1_7, A2_1_8, A2_3_4, A2_5_17)*, Cardio class (e.g. aerobics step aerobics body attack) *(A2_1_2, A2_1_5, A2_1_6, A2_3_1, A2_5_14),* Circuit training *(A2_1_3, A2_4_3, A2_5_7, A2_5_25)*, High intensity (e.g. HIT insanity) *(A2_1_4, A2_4_4, A2_5_18)*, Other fitness or exercise class *(A2_1_95, A2_5_21)*, Yoga *(A2_1_10, A2_3_6, A2_5_12),* Core strength *(A2_3_7)*, Pilates *(A2_1_9, A2_3_5, A2_5_11)*, Exercise machine *(A2_2_7, A2_5_2, A2_5_2_7)*, Rowing machine *(A1_3_7_2, A2_2_4, A2_5_2_4),* Exercise bike *(A2_2_2, A2_5_2_2)*, Cross training machine (e.g. Cross trainer SkiErg) *(A2_2_5, A2_5_2_1, A2_4_6, A2_5_8)*, Step machine *(A2_2_6, A2_5_2_5)*, Resistance weights machines *(A2_5_2_3)*, A session combining several gym or fitness machines or activities *(A2_2_1, A2_5_1)*, Free weights, resistance exercise, weight training (includes kettlebells and dumb-bells) *(A2_2_10, A2_2_11, A2_2_12, A2_2_13, A2_5_4)*, Weightlifting or powerlifting (using a barbell) *(A2_2_14, A2_2_8, A2_2_9, A2_5_6)*, Body weight exercises (e.g. pull ups press ups sit ups) *(A2_4_1, A2_5_3)*, Other weight training *(A2_2_95, A2_5_23)*, Aerial fitness *(A2_5_24)*, Obstacle course (e.g. Tough Mudder Spartan Rat Race) *(A1_8_7, A2_4_9)*, Cardio/aerobics at home *(A2_3_2, A2_3_3),* Exercise at home *(A2_3_9, A2_3_95),* Hula-hooping *(A2_4_12)*, Skipping *(A2_4_2, A2_5_5)*, Other fitness activities *(A2_4_95)*

**Running/jogging/athletics and multisports**: Track and field athletics *(A1_8_1),* Running or jogging *(A1_8_2)*, Fell or trail running *(A1_8_3)*, Running machine or treadmill *(A1_8_4, A2_5_2_6, A2_2_3)*, Triathlon (includes aquathlon and duathlon) *(A1_8_5)*, Modern Pentathlon *(A1_8_6)*

**Swimming and diving:** Swimming *(A1_7, A1_7_8*), Indoor swimming *(A1_7_1, A1_7_8_1)*, Outdoor pool swimming *(A1_7_2, A1_7_8_2)*, Open water swimming *(A1_7_3, A1_7_8_3)*, Diving *(A1_7_4)*,

**Other sports and leisure activities:** Climbing or mountaineering *(A1_3_1)*, Rock climbing or bouldering *(A1_3_1_1)*, Climbing or bouldering wall *(A1_3_1_2)*, Hill and mountain walking, hiking, mountaineering *(A1_3_1_3)*, Mountaineering and scrambling *(A1_3_1_4)*, Hill or mountain walking or hiking *(A1_3_1_5)*, Climbing or bouldering *(A1_3_1_6)*, Gliding, paragliding or handgliding *(A1_3_10)*, High ropes *(A1_3_19)*, Caving or pot holing *(A1_3_3)*, Abseiling *(A1_3_4)*, Orienteering *(A1_3_5)*, Parkour or free running *(A1_3_6)*, Hill or mountain walking or hiking *(Walk1_4)*, Canoeing or kayaking *(A1_3_11)*, Rafting *(A1_3_12)*, Water skiing or wakeboarding *(A1_3_13)*, Surfing, board surfing, body boarding, kite surfing, paddle boarding *(A1_3_14)*, Scuba diving or snorkelling *(A1_3_15)*, Life-saving *(A1_3_16)*, Sailing or windsurfing *(A1_3_17)*, Canoeing, kayaking or rafting *(A1_3_18)*, Rowing *(A1_3_7)*, Water based rowing *(A1_3_7_1)*, Sailing *(A1_3_8)*, Dinghy sailing *(A1_3_8_1)*, Yacht sailing *(A1_3_8_2)*, Keelboat sailing *(A1_3_8_3)*, Windsurfing *(A1_3_9)*, Other watersports such as dragon boat racing *(A1_3_95)*, Scuba diving or snorkelling *(A1_7_5)*, Life-saving *(A1_7_7)*, Horse riding *(A1_10)*, Hacking or pony trekking *(A1_10_1)*, Schooling *(A1_10_2)*, Show jumping *(A1_10_3)*, Dressage *(A1_10_4)*, Eventing *(A1_10_5)*, Other horse riding *(A1_10_6)*, Carpet bowls *(A1_11_1)*, Crown green bowls *(A1_11_2)*, Indoor flat green bowls *(A1_11_3)*, Outdoor flat green bowls *(A1_11_4)*, Short mat bowls *(A1_11_5)*, Boccia *(A1_11_6)*, Boules, petanque, deck bowls *(A1_11_7)*, Bowls *(A1_11_8)*, Flat green bowls (indoor/outdoor not specified) *(A1_11_9)*, Motorsports *(A1_14)*, Karting or go-karting *(A1_14_1)*, Motorcycle racing *(A1_14_2)*, Motorcar racing *(A1_14_3)*, Ice skating *(A1_13_4, A1_4_4)*, Skiing *(A1_6_1)*, Snowboarding *(A1_6_2)*, Sledding, luge, tobogganing *(A1_6_3)*, Curling *(A1_6_5)*, Ice skating *(A1_6_6)*, Other winter sports *(A1_6_7)*, Skiing or snowboarding *(A1_6_8),* Gymnastics *(A1_12_1)*, Trampolining *(A1_12_2)*, Garden trampolining *(A1_12_3)*, Cheerleading *(A1_12_4)*, Gymnastics or trampolining *(A1_12_5)*, Garden trampolining *(A1_4_7)*, Archery *(A1_4_2, A1_5_1)*, Fencing *(A1_4_3, A1_5_3)*, Boxing *(A1_5_2)*, Judo *(A1_5_4)*, Contact judo *(A2_5_4_1)*, Non-contact judo *(A2_5_4_2)*, Taekwondo *(A1_5_5)*, Martial arts *(A1_5_6)*, Karate *(A1_5_6_1)*, Tai chi *(A1_5_6_2, A1_5_9, A2_5_22, A2_1_11)*, Ju-Jitsu *(A1_5_6_3)*, Aikido *(A1_5_6_4)*, Chinese martial arts *(A1_5_6_5)*, Other martial arts *(A1_5_6_6)*, Shooting *(A1_5_7)*, Airgun (including pistol) *(A1_5_7_1)*, Rifle *(A1_5_7_2)*, Shotgun *(A1_5_7_3)*, Wrestling *(A1_5_8),* Rollerskating, inline skating, rollerblading *(A1_13_1)*, Skateboarding *(A1_13_3)*, Scootering *(Scoot1_1),* Angling or fishing *(A1_4_1)*, Coarse fishing *(A1_4_1_1)*, Game fishing *(A1_4_1_2)*, Sea fishing *(A1_4_1_3)*, Ten-pin bowling *(A1_4_10)*, Croquet *(A1_4_11)*, Snooker *(A1_4_12)*, Pool *(A1_4_13)*, Darts *(A1_4_14)*, Skittles *(A1_4_15)*, Cue based sports (e.g. billiards or combined snooker and pool answers) *(A1_4_16)*, Frisbee or ultimate frisbee *(A1_4_5)*, Other leisure activity or game *(A1_4_95, A_995)*, Creative or artistic dance *(Dance_1)*, Other types of dance *(Dance_4*, *Dance_6)*

**Gardening:** Gardening *(Act1_2, A_279)*


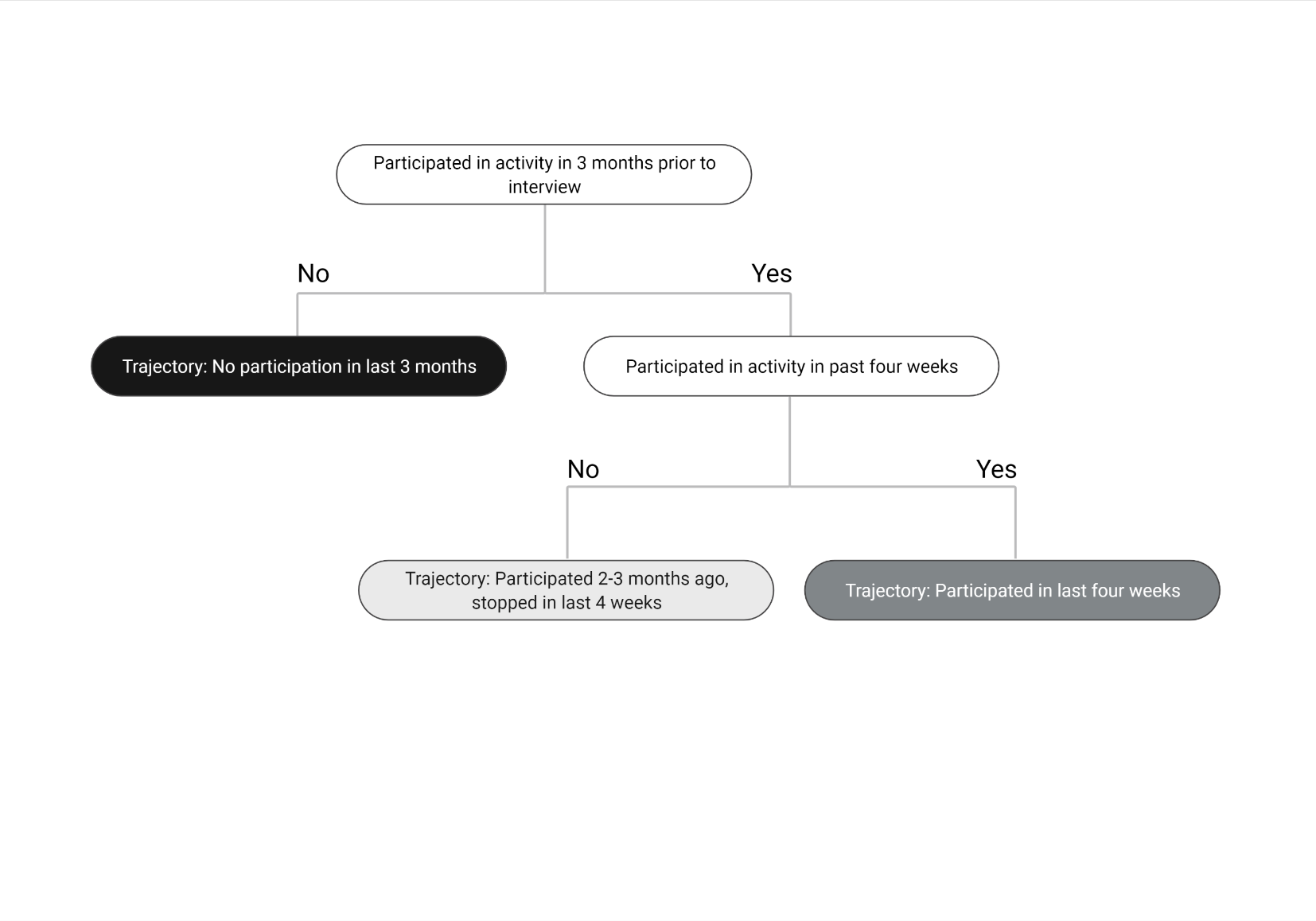
**Supplementary Figure 2. Within-person trajectory category derivation**

Created with biorender.com

*Note: It is not possible to determine if individuals who participated in an activity in the last four weeks also participated in the 2-3 months prior to interview.*

**Supplementary Figure 3A. Mean weekly durations by activity category for April-May respondents, by gender (n=74,430).**

**
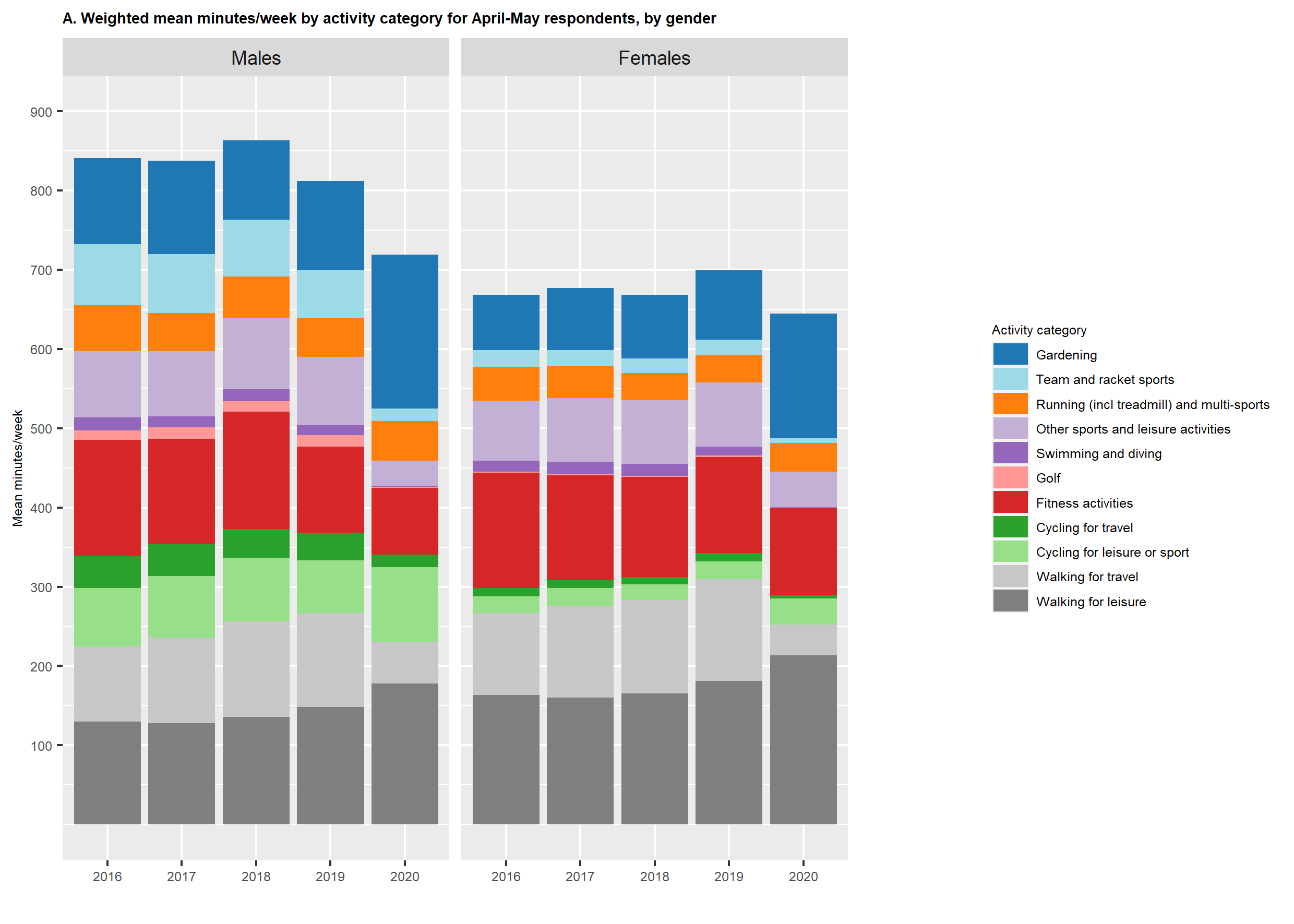
**

**Supplementary Figure 3B. Mean weekly durations by activity category for April-May respondents, by age (n=74,430).**

**
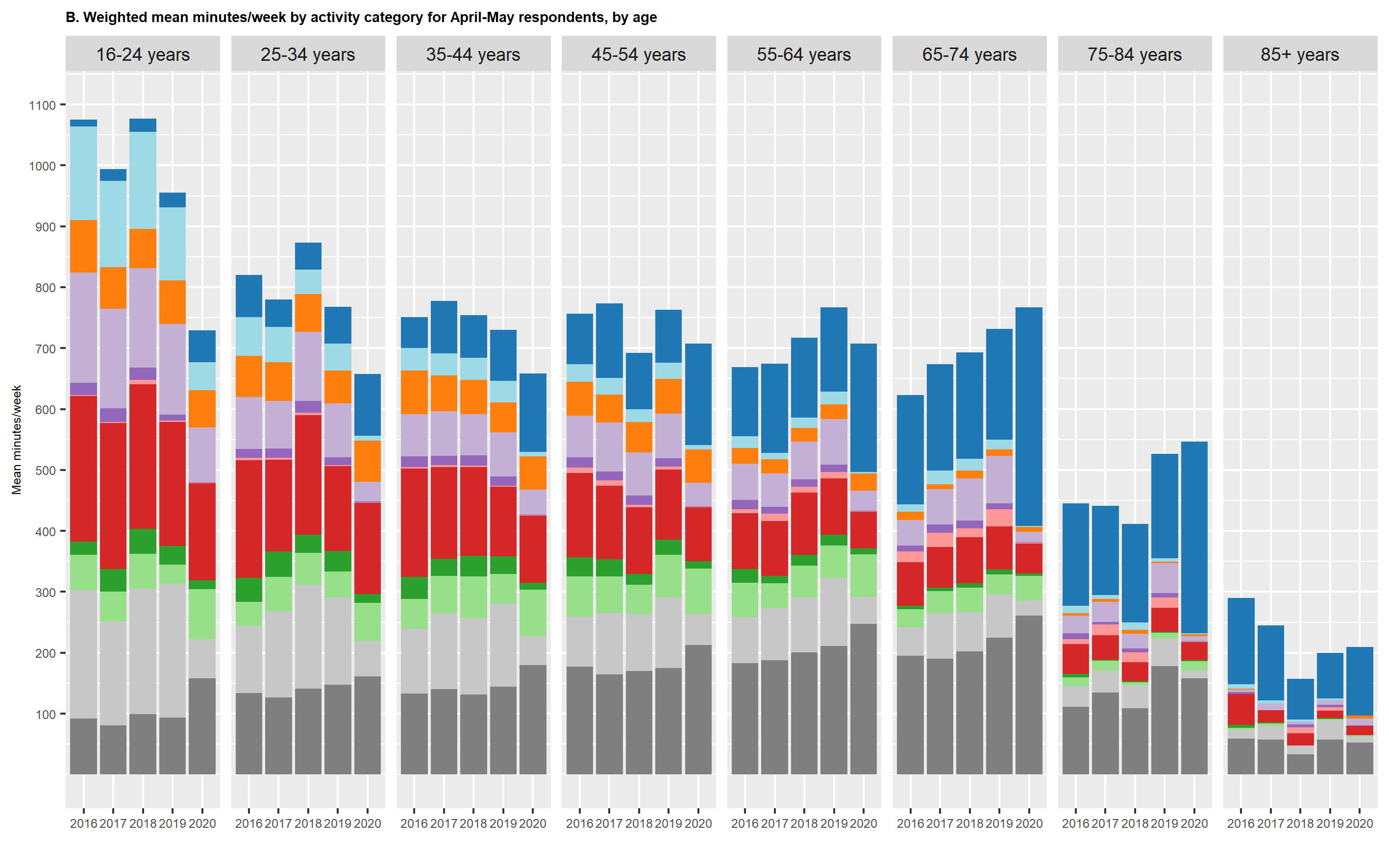
**

**Supplementary Figure 3C. Mean weekly durations by activity category for April-May respondents, by ethnicity (n=74,430).**


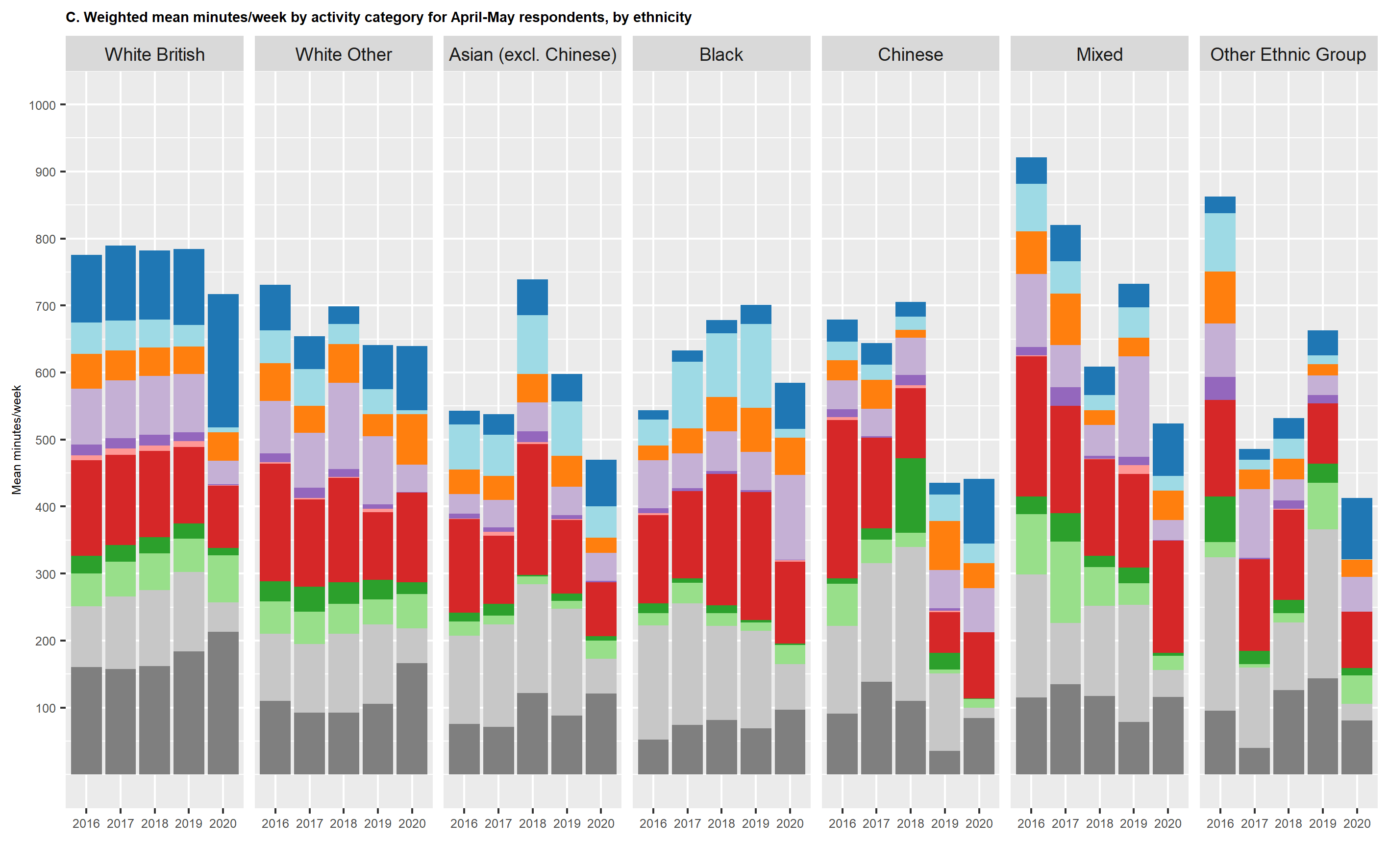


**Supplementary Figure 3D. Mean weekly durations by activity category for April-May respondents, by work status (n=74,430).**

**
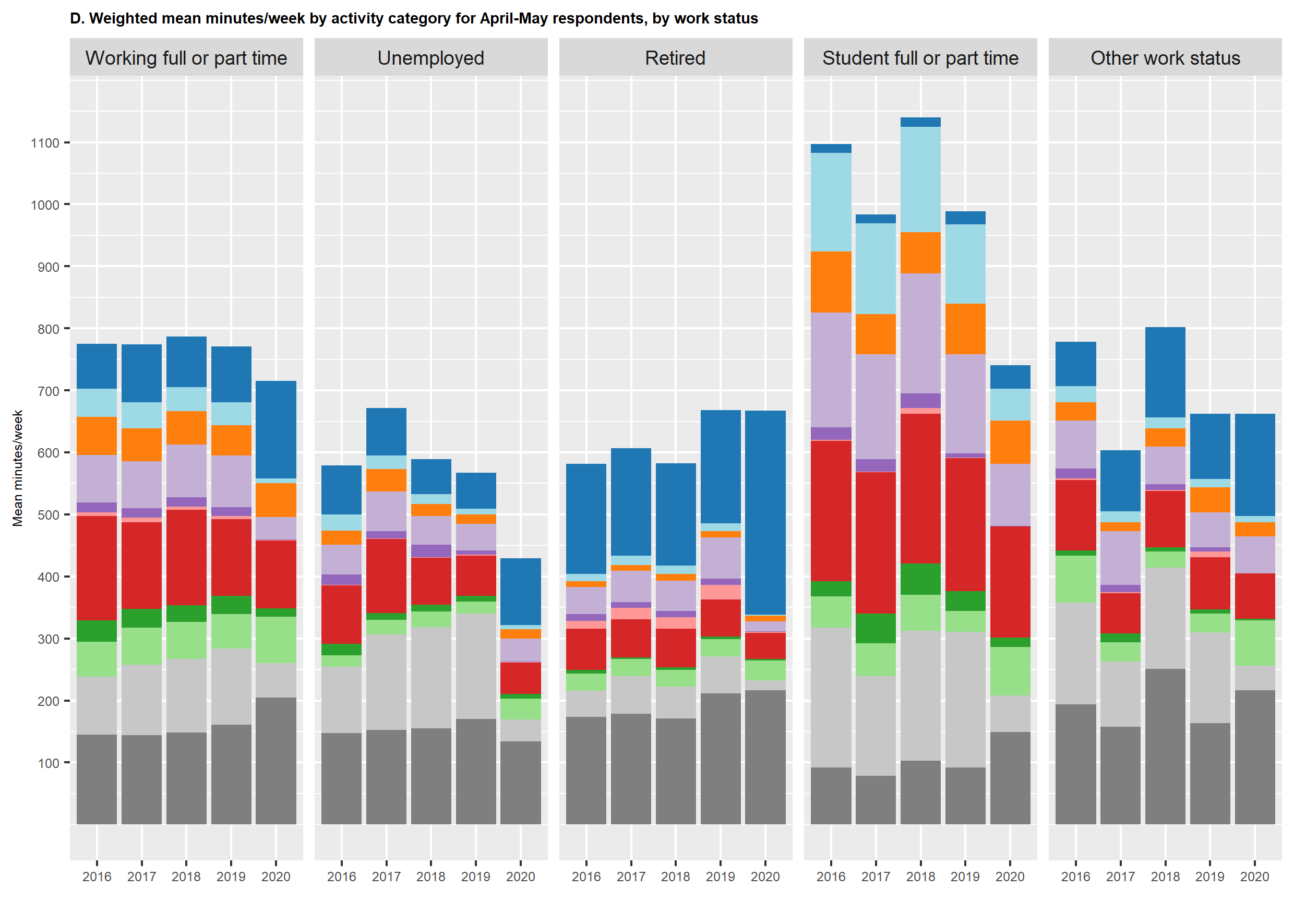
**

**Supplementary Figure 3E. Mean weekly durations by activity category for April-May respondents, by disability status (n=74,430).**

**
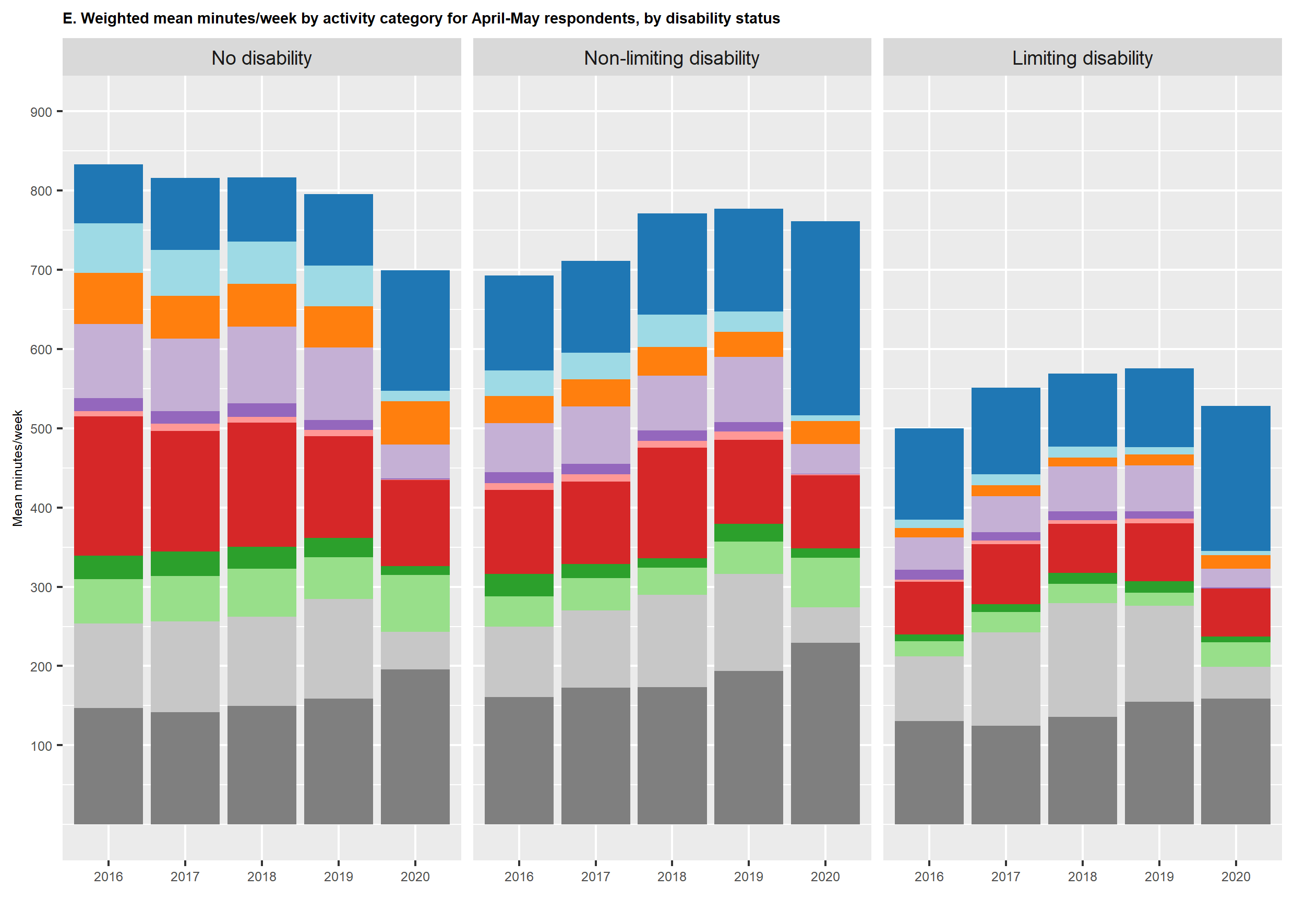
**

**Supplementary Figure 4A. Comparisons of levels of walking for leisure in April-May respondents in 2016-19 and 2020, by selected demographic subgroup, (n=74,430).**

**
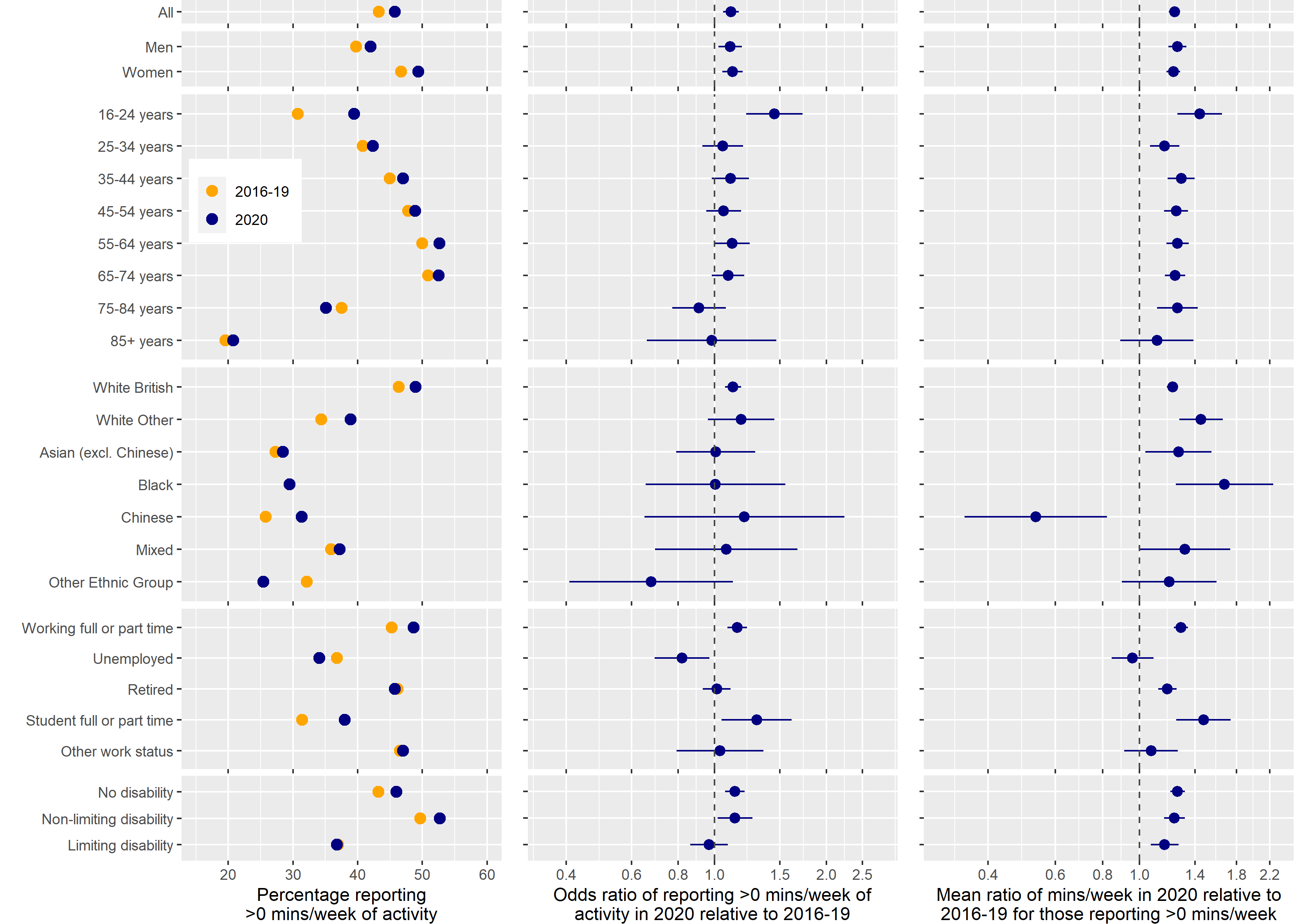
**

*Odds and mean ratios adjusted for age, sex, ethnicity, working status, disability status, NS SEC social class, education level, deprivation decile, children under 5 years in household, children 5-10 years in household, children 10-15 years in household, region, and urban-rural location.*


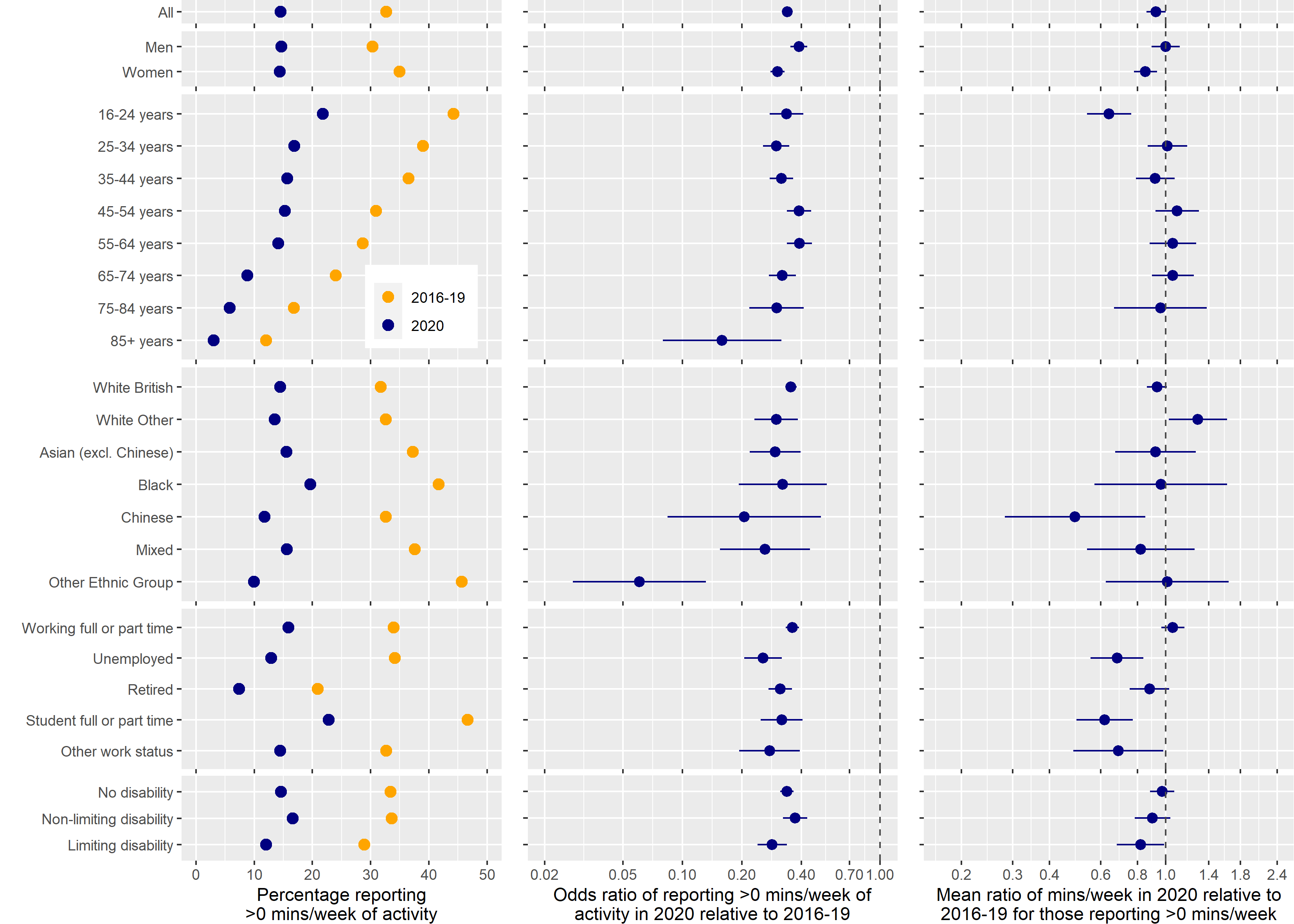
**Supplementary Figure 4B. Comparisons of levels of walking for travel in April-May respondents in 2016-19 and 2020, by selected demographic subgroup, (n=74,430).**

*Odds and mean ratios adjusted for age, sex, ethnicity, working status, disability status, NS SEC social class, education level, deprivation decile, children under 5 years in household, children 5-10 years in household, children 10-15 years in household, region, and urban-rural location. Data not shown when sample size <100.*

**Supplementary Figure 4C. Comparisons of levels of gardening in April-May respondents in 2016-19 and 2020, by selected demographic subgroup, (n=74,430).**

*
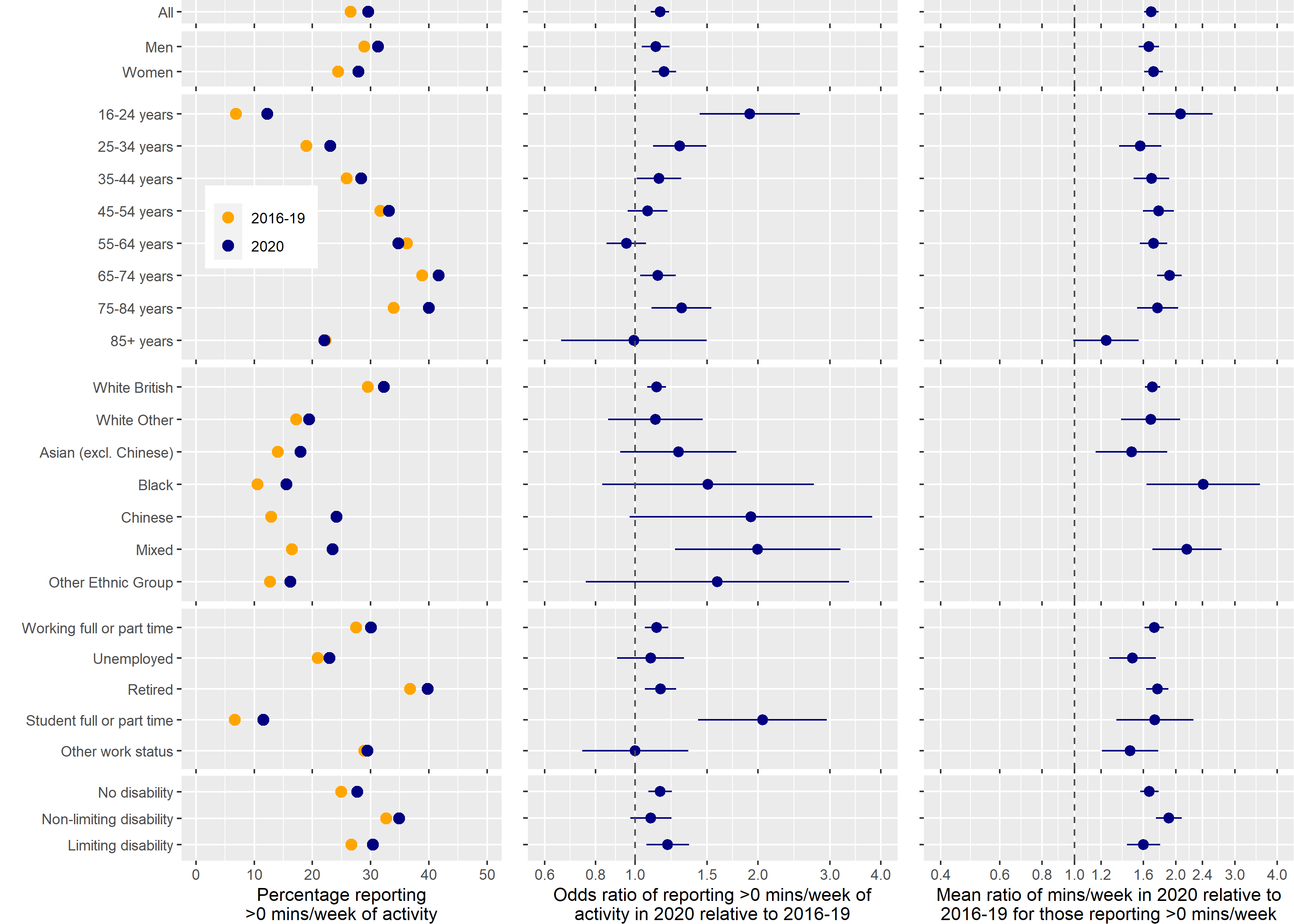
*

*Odds and mean ratios adjusted for age, sex, ethnicity, working status, disability status, NS SEC social class, education level, deprivation decile, children under 5 years in household, children 5-10 years in household, children 10-15 years in household, region, and urban-rural location. Data not shown when sample size <100.*

**Supplementary Figure 4D. Comparisons of levels of fitness activities in April-May respondents in 2016-19 and 2020, by selected demographic subgroup, (n=74,430).**

*
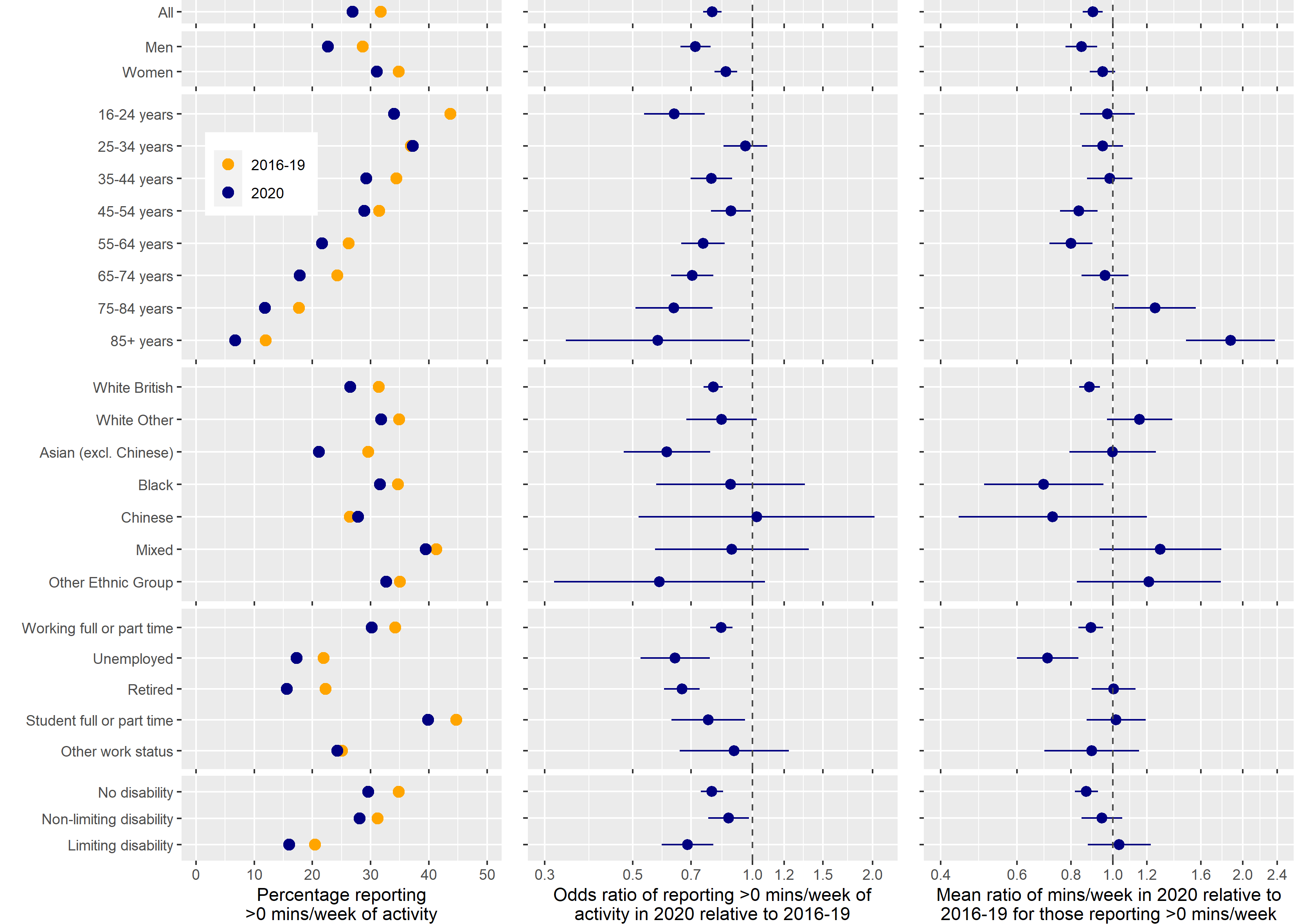
*

*Odds and mean ratios adjusted for age, sex, ethnicity, working status, disability status, NS SEC social class, education level, deprivation decile, children under 5 years in household, children 5-10 years in household, children 10-15 years in household, region, and urban-rural location.*

**Supplementary Figure 4E. Comparisons of levels of team and racket sports in April-May respondents in 2016-19 and 2020, by selected demographic subgroup, (n=74,430).**

**
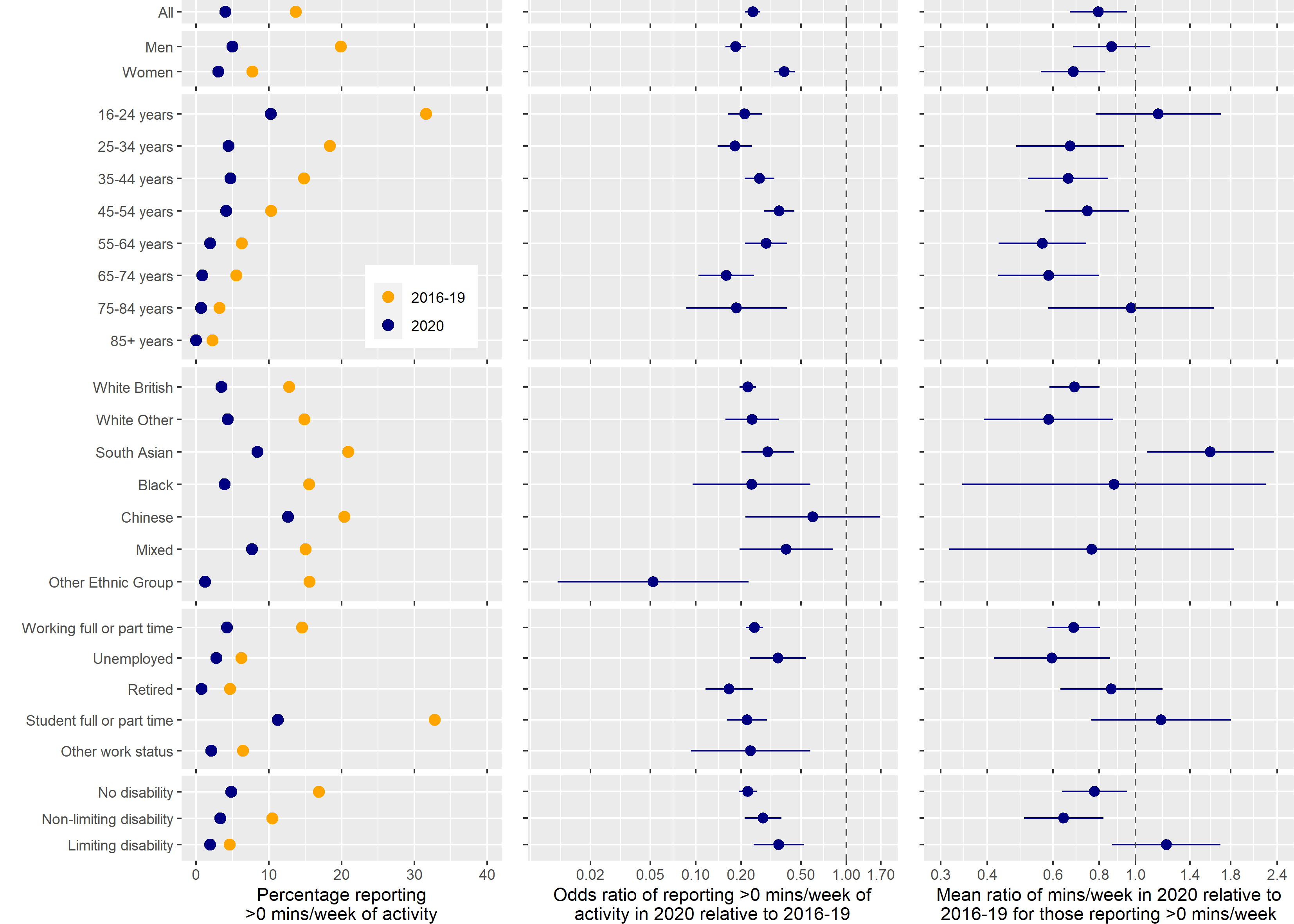
**

*Odds and mean ratios adjusted for age, sex, ethnicity, working status, disability status, NS SEC social class, education level, deprivation decile, children under 5 years in household, children 5-10 years in household, children 10-15 years in household, region, and urban-rural location. Data not shown when sample size <100.*
